# Injectable Mn–Icariin-functionalized silk fibroin/PEG hydrogels restore redox homeostasis and reprogram osteogenic–angiogenic coupling for diabetic bone regeneration

**DOI:** 10.1093/rb/rbag143

**Published:** 2026-07-03

**Authors:** Yukai Huang, Shihao Zhang, Xiaogang Zhou, Lang Qin, Zeng Lin, Binghuang Yao, Zhiyang Gao, Huanxin He, Jinwang Huang, Jie Peng, Huizhen Du, Dingxin Wang, Junyuan Zeng, Dihan Su, Zenggan Chen, Yulin Li, Libo Jiang, Mingdong Zhao

**Affiliations:** Department of Orthopaedics, Jinshan Hospital, Fudan University, Shanghai 201508, China; Key Laboratory for Ultrafine Materials of Ministry of Education, Frontiers Science Center for Materiobiology and Dynamic Chemistry, Research Center for Biomedical Materials of Ministry of Education, School of Materials Science and Engineering, East China University of Science and Technology, Shanghai 200237, China; Department of Orthopaedic Surgery, Zhongshan Hospital, Fudan University, Shanghai 200032, China; Key Laboratory for Ultrafine Materials of Ministry of Education, Frontiers Science Center for Materiobiology and Dynamic Chemistry, Research Center for Biomedical Materials of Ministry of Education, School of Materials Science and Engineering, East China University of Science and Technology, Shanghai 200237, China; Department of Orthopaedic Surgery, Zhongshan Hospital, Fudan University, Shanghai 200032, China; Department of Orthopaedics, Jinshan Hospital, Fudan University, Shanghai 201508, China; Department of Orthopaedics, Jinshan Hospital, Fudan University, Shanghai 201508, China; Department of Orthopaedics, Jinshan Hospital, Fudan University, Shanghai 201508, China; Department of Orthopedic Surgery, Zhongshan Hospital (Xiamen), Fudan University, Xiamen 361015, China; Xiamen Osteoporosis Specialized Prevention and Treatment Center, Xiamen 361000, China; Clinical Medical College of Xiamen Medical College, Xiamen 361023, China; Department of Orthopaedic Surgery, Zhongshan Hospital, Fudan University, Shanghai 200032, China; Department of Orthopaedics, Jinshan Hospital, Fudan University, Shanghai 201508, China; Department of Orthopaedics, Jinshan Hospital, Fudan University, Shanghai 201508, China; Department of Orthopaedic Surgery, Zhongshan Hospital, Fudan University, Shanghai 200032, China; Department of Orthopaedic Surgery, Zhongshan Hospital, Fudan University, Shanghai 200032, China; Department of Orthopedic Surgery, Zhongshan Hospital (Xiamen), Fudan University, Xiamen 361015, China; Xiamen Osteoporosis Specialized Prevention and Treatment Center, Xiamen 361000, China; Key Laboratory for Ultrafine Materials of Ministry of Education, Frontiers Science Center for Materiobiology and Dynamic Chemistry, Research Center for Biomedical Materials of Ministry of Education, School of Materials Science and Engineering, East China University of Science and Technology, Shanghai 200237, China; Department of Orthopaedic Surgery, Zhongshan Hospital, Fudan University, Shanghai 200032, China; Department of Orthopedic Surgery, Zhongshan Hospital (Xiamen), Fudan University, Xiamen 361015, China; Xiamen Osteoporosis Specialized Prevention and Treatment Center, Xiamen 361000, China; Department of Orthopaedics, Jinshan Hospital, Fudan University, Shanghai 201508, China

**Keywords:** oxidative stress, diabetic bone defect repair, silk fibroin, bioactive hydrogel, M2 macrophages

## Abstract

Bone defect repair under hyperglycemic conditions is limited by sustained oxidative stress, chronic low-grade inflammation and disrupted osteoimmune-vascular coupling. In this study, we constructed an injectable and biodegradable silk fibroin/PEG hydrogel incorporating MnO_2_ honeycomb nanospheres and liposome-encapsulated icariin (FG/Mn@Ica) to rebuild a bioactive and mechanocompatible microenvironment for diabetic bone regeneration. The hydrogel presented an interconnected porous morphology, elastic-dominant rheology, adaptive compressive behavior and controlled degradation with the sustained release of Mn ions and Ica. Functionally, FG/Mn@Ica reprograms the high-glucose microenvironment at multiple levels. *In vitro*, it enhances the proliferation of bone marrow mesenchymal stem cells and osteogenic commitment while simultaneously attenuating reactive oxygen species accumulation, stabilizing the mitochondrial membrane potential and activating the AMPK–SIRT1–NRF2-mediated antioxidant axis. Moreover, FG/Mn@Ica promoted the migration of human umbilical vein endothelial cells (HUVECs) and angiogenic tube formation, induced the polarization of pro-regenerative M2 macrophages with elevated Arg1, and suppressed senescence-associated and inflammation-associated markers. *In vivo*, in a rat femoral defect model, FG/Mn@Ica significantly promoted new bone regeneration. Overall, FG/Mn@Ica integrates mechanical support, restoration of redox homeostasis, osteoimmune modulation and osteogenic reinforcement, offering a rational biomaterial strategy for bone regeneration under diabetic stress.

## Graphical abstract

**Graphical Abstract:**
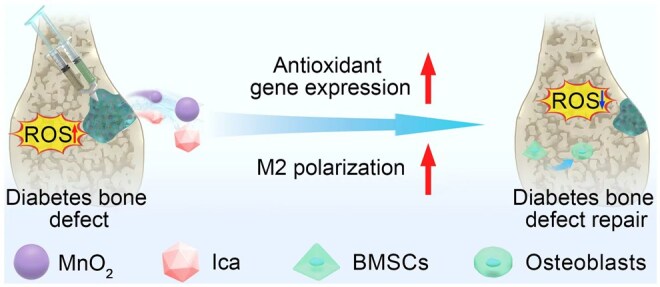

## Introduction

Achieving effective bone regeneration in hyperglycemic settings remains a major clinical practice since diabetes often leads to the disruption of bone remodeling, delayed healing, and a greater incidence of fracture non-union [1, 2]. Poor regenerative outcomes are driven not only by insufficient osteogenic capacity but also by a profoundly dysfunctional microenvironment characterized by elevated oxidative stress, sustained inflammatory responses, mitochondrial dysfunction and premature cellular senescence [3–5]. Collectively, these changes compromise osteoblast–osteoclast coupling and angiogenesis, suppress the osteogenic commitment of bone marrow mesenchymal stem cells (BMSCs), and ultimately hinder bone regeneration [6–8]. Consequently, therapeutic strategies that rely solely on structural filling or osteoinductive cues are insufficient; instead, the reconstruction of a pro-regenerative biochemical and immunological niche is essential for effective bone repair under diabetic stress [9, 10].

Among the pathological factors implicated in diabetic bone degeneration, the persistent accumulation of reactive oxygen species (ROS) represents a central driving force. Hyperglycemia-induced overproduction of ROS destabilizes the mitochondrial membrane potential, activates senescence-associated signaling pathways and downregulates osteogenic transcriptional programs in BMSCs [11, 12]. In parallel, the imbalance of osteoimmune regulation, particularly the reduced polarization of pro-regenerative M2 macrophages and persistent inflammatory signaling, further aggravates tissue damage and delays bone healing [13, 14]. Collectively, these findings highlight the necessity of simultaneously restoring redox homeostasis, modulating immune responses and supporting osteogenic differentiation [15]. Given these pathological constraints, increasing attention has been directed toward the development of bioactive biomaterials that not only fill bone defects but also actively reshape the diseased microenvironment [16]. Specifically, microenvironment-responsive hydrogels have emerged as attractive candidates owing to their injectability, tissue conformability and capacity to serve as multifunctional carriers for therapeutic ions and small molecules [5, 17]. Moreover, bioactive phytochemicals and ion-releasing components have shown promise in enhancing osteogenic differentiation, facilitating angiogenesis and improving bone-immune crosstalk [9, 18]. However, in diabetic bone repair, most existing hydrogels target only one pathway—scavenging ROS, suppressing inflammation or promoting osteogenesis; thus, materials that can simultaneously regulate redox balance, tune osteoimmune responses and promote osteogenesis while maintaining suitable mechanics are still lacking, motivating the design of the present hydrogel system.

In light of these considerations, protein–polymer hybrid hydrogels have garnered increasing attention, as they provide mechanically compliant and porous 3D architectures that accommodate irregular defect geometries while supporting cell infiltration, nutrient diffusion and programmable degradation [18, 19]. Within this framework, silk fibroin (SF) and polyethylene glycol (PEG) hybrid hydrogels offer an elastic-dominant and tissue-adaptable matrix that can function as an effective reservoir for bioactive cues within the defect microenvironment [20, 21]. From a functional perspective, MnO_2_-based nanomaterials have emerged as potent redox-responsive components that can participate in cellular ROS scavenging, stabilizing mitochondrial function and activating endogenous antioxidant signaling pathways [22]. Simultaneously, icariin, a well-characterized natural flavonoid, has shown considerable potential for promoting osteogenic differentiation, enhancing angiogenesis, and modulating osteoimmune responses, particularly under metabolically compromised or inflammation-compromised conditions [23–25].

**Figure 1:**
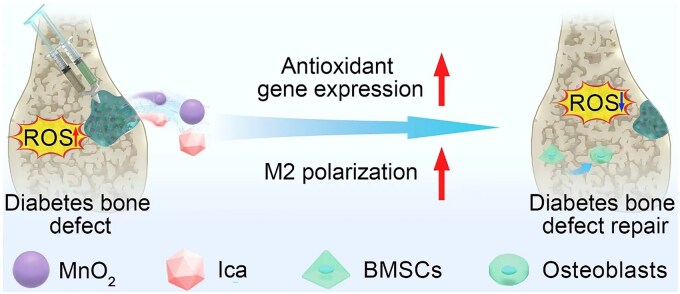
Schematic overview of FG/Mn@Ica hydrogel–mediated regulation of the diabetic bone microenvironment, highlighting sustained MnO_2_ and Ica release, antioxidant activation, immune modulation, and enhanced osteogenesis leading to improved bone regeneration.

Herein, we engineered an injectable and biodegradable SF/PEG hydrogel in which Mn-based redox-regulating nanoparticles serve to mitigate hyperglycemia-induced oxidative stress and restore mitochondrial homeostasis, whereas icariin-loaded nanocarriers provide osteogenic, angiogenic and osteoimmune reinforcement within the defect microenvironment (Figure 1). This integrated platform synergistically couples mechanical compliance with coordinated regulation of redox balance, inflammatory and senescence-associated signaling, and osteogenic–angiogenic coupling, thereby reprogramming the diabetic bone microenvironment toward a pro-regenerative state. Consequently, the hydrogel robustly promoted osteogenesis, enhanced angiogenic activity and ultimately facilitated effective bone regeneration.

**Figure 2:**
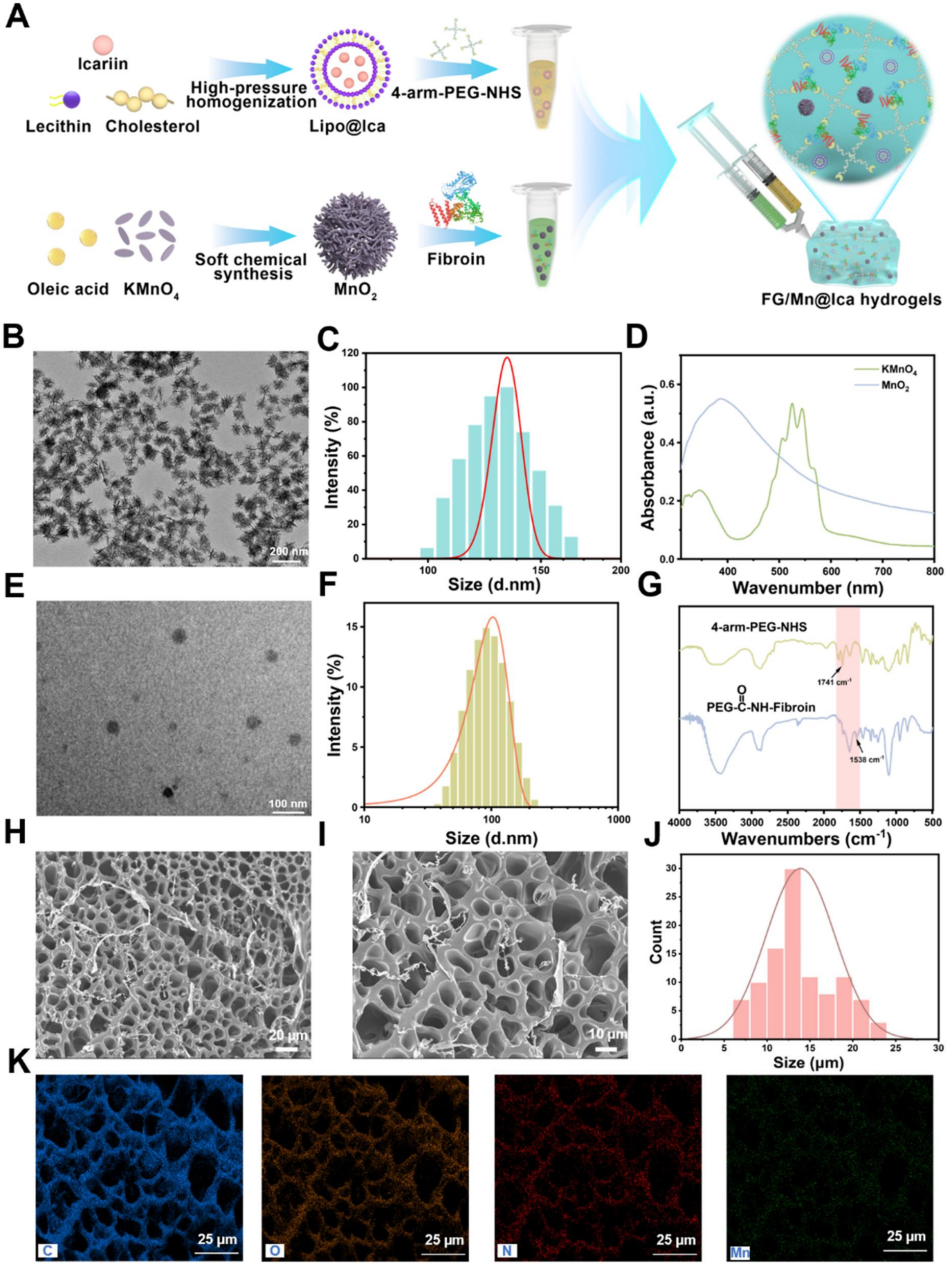
Preparation and structure of the FG/Mn@Ica hydrogel. (**A**) Schematic diagram of the FG/Mn@Ica hydrogel preparation. (**B**) TEM image of the MnO_2_ nanoparticles. (**C**) Particle size distribution of the MnO_2_ nanoparticles. (**D**) UV-vis absorption spectra of the KMnO_4_ solution and MnO_2_. (**E**) TEM image of the Liposome@Ica nanoparticles. (**F**) Particle size distribution of the Liposome@Ica nanoparticles. (**G**) FT-IR spectrum of the FG/Mn@Ica hydrogels. (**H**–**J**) SEM image and pore size distribution of the FG/Mn@Ica hydrogel. (**K**) Distribution of the elements in the FG/Mn@Ica hydrogel.

## Materials and methods

### Materials

4-arm poly(ethylene glycol) succinimidyl ester (4-arm PEG-NHS, Mw = 10 kDa) was obtained from Xiamen Sinopeg Biotechnology Co., Ltd. Lecithin (High Potency) was supplied by Sinopharm Chemical Reagent Co., Ltd., while oleic acid (≥99%) and cholesterol were sourced from Shanghai Titan Scientific Co., Ltd. Icariin was provided by MedChemExpress. Potassium permanganate (KMnO_4_) and ethanol were purchased from Sinopharm Chemical Reagent (Shanghai, China). Lithium bromide (LiBr) and PEG were obtained from Shanghai Aladdin Biochemical Technology Co., Ltd. α-MEM, fetal bovine serum and penicillin–streptomycin were purchased from Gibco (MA, USA). Experimental rats were supplied by SPF Biotechnology Co., Ltd. (Beijing, China). All reagents and chemicals were used directly without additional purification. Primary antibodies against AMPK, HO-1, NQO-1, Arg1, GAPDH and β-Tubulin were obtained from Proteintech (Chicago, USA). Antibodies targeting OPN, OCN, NRF2 and TNF-α were purchased from Abclonal (MN, USA), and the p-AMPK antibody was obtained from Selleck (Shanghai, China). Antibodies against SIRT1, SOD1, SOD2, P16, P21 and P53 were obtained from Zen-Bio (Chengdu, China). Antibodies against collagen I and RUNX2, as well as flow-cytometry antibodies CD11b and CD206, were purchased from Abcam (MA, USA). The CCK-8 kit, ALP staining kit, Alizarin Red S (ARS) staining kit, live/dead viability kit, Ki-67 immunofluorescence kit, JC-1 staining kit and MitoSOX Red kit were supplied by Beyotime (Beijing, China).

### Preparation of Liposome@Ica nanoparticles

All nanoparticles were freshly prepared before use. Liposome@Ica nanoparticles were prepared using a high-pressure homogenizer. In brief, 240 mg of soybean lecithin, 60 mg of cholesterol and 24 mg of icariin were dispersed and dissolved in 4 mL of 50°C hot ethanol. This dispersion was then added to 20 mL of ultrapure water and introduced into a high-pressure homogenizer. Initial shear homogenization was performed at 19 000 rpm for 5 min, followed by high-pressure homogenization at 40 MPa for 5 min to yield the Liposome@Ica nanoparticle dispersion.

### Preparation of MnO_2_ nanoparticles

KMnO_4_ (0.199 g) was first dissolved in 100 mL of deionized water under vigorous stirring. After approximately 1 h of continuous stirring, oleic acid (1.78 g) was introduced into the solution. The mixture was then kept at room temperature for 5 h, during which a brown-black product gradually formed. The resulting precipitates were collected by centrifugation (12 000 rpm, 15 min), followed by two washing cycles with distilled water and ethanol to eliminate unreacted species. The obtained MnO_2_ nanoparticles were finally redispersed in water and lyophilized for storage [26].

### Preparation of FG/Mn@Ica hydrogels

The SF solution was generated via degumming and dissolution of Bombyx mori silk fibers using a previously established protocol [27]. To prepare the hydrogel precursors, solution A was formulated by dissolving 4-arm PEG-NHS (10%, w/v) in the Liposome@Ica nanoparticle dispersion. Solution B was prepared by dispersing MnO_2_ nanoparticles (0.5 mg/mL) into a 10% (w/v) SF solution. Equal volumes of solutions A and B were subsequently co-injected into a mold using a dual-syringe system, where *in situ* crosslinking yielded the SF-PEG composite hydrogel (FG/Mn@Ica).

### Characterization of Liposome@Ica and MnO_2_ nanoparticles

Take the Liposome@Ica nanoparticle dispersion solution (concentration 0.1 mg/mL), place two drops onto a copper grid, and dry at room temperature for several hours. Liposome@Ica morphology was examined by transmission electron microscopy (TEM) (1400 kV), and particle size analysis was performed with a Malvern laser particle size analyzer at 0.1 mg/mL and 25°C. MnO_2_ nanoparticles were characterized using the same protocol. The zeta potentials of MnO_2_ nanoparticles and Liposome@Ica were measured by dynamic light scattering.

### Fourier transform infrared spectroscopy

A 2-mg sample (water-containing samples were freeze-dried) was weighed, and signals were collected via lithium bromide pressure-sheet analysis with wave numbers ranging from 4000 to 400 cm^−1^. All spectra were processed with CO_2_ and H_2_O reduction, noise removal and baseline correction.

### Surface morphology of hydrogels

Scanning electron microscopy (SEM, JSM-IT800, Japan) coupled with energy-dispersive spectroscopy (EDS) was employed to characterize the surface morphology and microstructural features of the hydrogels. Sample preparation for imaging involved freeze-drying followed by gold sputter-coating of the fracture surfaces to enhance conductivity. The internal porous structure was visualized by SEM, and pore size distribution was quantified in ImageJ.

### Rheological performance testing

Rheological measurements were conducted according to procedures reported in our previous work [28]. Briefly, rheological experiments were performed at 25 ± 1°C using a HAAKE RS600 rheometer (Thermo Fisher Scientific). Frequency (0.1–100 Hz) and temperature (25–80°C, 1% strain) sweep tests were conducted to assess the rheological properties.

### Mechanical property evaluation

A universal testing machine (CMT-2503, Metter, China) was employed to evaluate the mechanical performance of the hydrogels at room temperature. Hydrogel samples were molded into cylindrical specimens (diameter 15 mm × thickness 7 mm) and tested for compression at a rate of 5 mm/min using a 20 N mechanical sensor. Data acquisition generated stress–strain curves [28].

### H_2_O_2_-scavenging assay of MnO_2_ nanoparticles

The ROS-eliminating capability of MnO_2_ nanoparticles was detected by quantifying the residual H_2_O_2_ after incubating with nanoparticles for specific times. Briefly, 40 µL MnO_2_ with 1 µg/mL concentration was added to a 360 µL H_2_O_2_ (10 × 10^−6^ M) solution, and the residual H_2_O_2_ was determined with the H_2_O_2_ assay kit after being incubated for 0, 1, 2, 4, 8 and 10 h.

### Degradability and release testing

To evaluate the degradation and release profiles of the hydrogels, 1 mL of hydrogel was immersed in 10 mL of phosphate-buffered saline (PBS) and maintained at 37°C. At scheduled time intervals, the samples were removed, blotted and weighed, and the percentage of mass loss was calculated according to the following formula:


(1) $$\mathrm{weight}\;\mathrm{loss}\;(\%)=\frac{W_{0}-W_{n}}{W_{0}}\times 100\%,$$

where $W_{0}$ represents the initial mass of the undegraded hydrogel and $W_{n}$ denotes the sample mass at each degradation time point.

The concentrations of Ica and Mn ions were quantified using spectrophotometry and high-performance liquid chromatography-inductively coupled plasma mass spectrometry (HPLC-ICP-MS, NexION 2000-A10, USA), respectively.

### Swelling test of hydrogels

For swelling characterization, the fabricated hydrogels were lyophilized to obtain their dry weight *W*_0_. These samples were separately immersed in DI water, and their weights were monitored until no significant change was observed to obtain the equilibrium swelling weight *W_t_*. The experiments were performed in triplicate under the same conditions, and the swelling ratio was calculated by Equation (2):


(2) $$\mathrm{swelling}\;\mathrm{ratio}\;(\%)=\frac{W_{t}-W_{0}}{W_{0}}\times 100\%.$$

### Cytocompatibility and hemocompatibility evaluation of hydrogels

Extract-based assays were employed to assess the cytocompatibility of the hydrogels. Sterilized hydrogels were incubated in α-MEM (10% FBS, 1% penicillin–streptomycin) at 37°C for 24 h to obtain hydrogel extracts, which were collected, centrifuged and used as extraction media. BMSCs and HUVECs were allowed to adhere overnight and subsequently cultured with the corresponding extracts. Cell viability was evaluated by CCK-8 assay at days 1, 3 and 7, and live/dead (Calcein-AM/PI) staining was performed in parallel and imaged by fluorescence microscopy. Ki-67 immunofluorescence staining was used to assess proliferation, and Ki-67-positive cell ratios were quantified in ImageJ. Hemocompatibility was examined by a direct-contact hemolysis test, in which hydrogels were incubated with a red blood cell suspension at 37°C, followed by centrifugation and absorbance measurement at 540 nm. Saline and deionized water served as negative and positive controls, and hemolysis rates <5% were considered acceptable.

### Osteogenic differentiation of BMSCs *in vitro*

Sterilized hydrogels were incubated in high-glucose (HG) osteogenic medium (α-MEM containing 10% FBS, 0.05 mM vitamin C, 10 mM β-glycerophosphate, 10 nM dexamethasone and 25 mM glucose) at 37°C for 24 h to obtain hydrogel extracts under diabetic-like osteogenic conditions. After overnight culture, BMSCs were exposed to the corresponding extracts, with medium renewal every 3 days. Alkaline phosphatase (ALP) activity and matrix mineralization were evaluated by BCIP/NBT staining (days 7 and 14) and Alizarin Red S staining (days 14 and 21), respectively. BMSCs treated with the extracts for 7 days were lysed in RIPA buffer containing PMSF, and protein concentrations were measured by BCA assay. Equal amounts of protein were separated by SDS-PAGE, transferred to PVDF membranes, and incubated with primary antibodies against RUNX2, OPN, OCN and β-actin, followed by HRP-conjugated secondary antibodies. Bands were visualized using ECL and quantified in ImageJ. Confocal microscopy was used to visualize immunofluorescence signals of Collagen I and RUNX2 following extract treatment, enabling qualitative and quantitative evaluation.

### Cell migration and angiogenesis assays under hydrogel–extract stimulation

Sterilized hydrogels were incubated in HG medium at 37°C for 24 h to obtain hydrogel–HG extracts, which were filtered and used for subsequent cell assays. For the scratch-wound assay, confluent HUVECs and BMSCs in 6-well plates were scratched with a sterile 200-μL pipette tip, rinsed with PBS, and cultured in hydrogel–HG extracts. Images at 0, 12 and 24 h were acquired by an inverted microscope, and wound closure was quantified using ImageJ. For the transwell migration assay, HUVECs were seeded in the upper chambers (8-μm inserts) in serum-free HG medium, while the lower chambers contained hydrogel-HG extracts with 10% FBS as a chemoattractant. Migrated cells were fixed, crystal-violet stained, imaged and quantified using ImageJ. For the tube-formation assay, HUVECs (1 × 10^5^ cells/well) were seeded on Matrigel-coated 96-well plates and cultured with hydrogel-HG extracts. Capillary-like networks were visualized by inverted fluorescence/phase-contrast microscopy and angiogenic parameters were analyzed using the Angiogenesis Analyzer plugin.

### Oxidative stress and antioxidant pathway analysis

Intracellular ROS levels in BMSCs were evaluated using a DCFH-DA probe (Beyotime, China) following treatment with hydrogel-HG extracts. After incubation with DCFH-DA at 37°C for 20-30 min and PBS washing, fluorescence and flow cytometry were performed, and ROS intensity/positive cell ratios were quantified using ImageJ and FlowJo. Changes in mitochondrial membrane potential (ΔΨm) were assessed by JC-1 staining, with the red-to-green fluorescence ratio used as an indicator of ΔΨm status. BMSCs exposed to hydrogel-HG extracts were lysed in RIPA buffer containing PMSF, and total protein content was measured by BCA assay. Following SDS-PAGE separation and transfer onto PVDF membranes, equal protein samples were immunoblotted using primary antibodies against AMPK, p-AMPK, SIRT1, NRF2, HO-1, NQO-1, SOD1, SOD2 and β-tubulin, with HRP-conjugated secondary antibodies applied thereafter. Bands were visualized by ECL and quantified in ImageJ to analyze antioxidant and inflammation-related pathway activation.

### Macrophage polarization and senescence-associated assays

Flow cytometric analysis of CD206 expression was performed to determine M2 polarization in bone marrow-derived macrophages (BMDMs) following extract treatment, with data analyzed in FlowJo. Arg1 expression in BMDMs was further examined by Western blot. For senescence-associated protein analysis, BMSCs cultured in HG medium and exposed to hydrogel-HG extracts were collected for Western blot detection of P16, P21, P53 and TNF-α, using β-actin as a loading control. Protein bands were detected by ECL and quantified in ImageJ to evaluate hydrogel-mediated regulation of macrophage polarization and senescence-related inflammatory signaling.

### *In vivo* femoral defect model and bone regeneration evaluation

All animal procedures followed ARRIVE guidelines and were approved by the Animal Care and Use Committee of Zhongshan Hospital, Fudan University (Shanghai, China) (Ethics approval number: #2024-023). Bone defects were created in the distal femoral condyle under sterile conditions and filled with the corresponding hydrogels, while the blank group received no implantation. At 6 and 12 weeks post-implantation, rats were euthanized and femurs were collected for further analyses. Micro-CT scanning was performed to generate 3D reconstructions of the defect area. Bone mineral density (BMD), bone volume fraction (BV/TV) and trabecular thickness (Tb.Th) were quantified within the region of interest. To evaluate dynamic bone formation, undecalcified sections were prepared, and double fluorescent labeling with calcein and alizarin red was observed on the bone sections, which were imaged to calculate the mineral apposition rate (MAR). For histological evaluation, the harvested femurs were fixed, decalcified, embedded in paraffin and sectioned into serial slices. The sections were subsequently subjected to H&E, toluidine blue, van Gieson and Masson’s trichrome staining to examine tissue morphology, collagen matrix deposition, and the extent of new bone formation and defect repair.

### Statistical analysis

All experimental data are presented as mean ± standard deviation (SD). Statistical analysis was conducted using SPSS 20.0 software. Comparisons among multiple groups were performed using one-way analysis of variance, followed, when necessary, by *post hoc* multiple-comparison tests. Differences were considered statistically significant at *P < 0.05, highly significant at **P < 0.01, and extremely significant at ***P < 0.001.

## Results and discussion

### Preparation and structural characterization of the FG/Mn@Ica hydrogel

Sericin, owing to its excellent biocompatibility and processability, finds extensive application in the biomedical field. In recent research on sericin hydrogels, Zheng et al. developed a sericin/magnesium oxide (SF/MgO) composite scaffold for irregular bone defect regeneration [29]. The Kovacina team prepared SF-Ca hydrogels by adding Ca^2+^ to the sericin solution and employing a Ru(III)/SPS photoinitiation system for photopolymerization [30]. However, existing sericin hydrogels suffer from issues including crosslinker residue, conflicting demands for crosslinking efficiency and structural controllability, limited functionality, complex preparation and inability to mimic the multi-regional, multi-level architecture of natural tissues. In this experimental approach, we capitalized on the highly efficient reaction between 4-arm PEG-NHS and sericin amino groups to develop a sericin hydrogel that combines rapid preparation, long-term stability, and multifunctional integration. This approach represents a key breakthrough in addressing the aforementioned challenges.

The FG/Mn@Ica hydrogels were fabricated by simply mixing two components of 4-arm-PEG-NHS and fibroin solutions. MnO_2_ honeycomb nanospheres were synthesized via a simple soft chemical route at room temperature, as described in Figure 2A and Supplementary Figure S1 [31]. At appropriate concentrations, oleic acid forms a stable oil-in-water (O/W) emulsion where KxMnO_2_ reacts with oleic acid via the ‘Baeyer test for unsaturation’ at the interface, generating KxMnO_2_ nuclei. Low precursor concentrations lead to sparse lamellar platelets and a fragile shell, which collapses upon ethanol removal of oleic acid and cis-diol, producing honeycomb nanospheres [31]. A typical TEM image of honeycomb-like MnO_2_ nanospheres is illustrated in Figure 2B. The nanospheres had radially aligned lamellae perpendicular to the surface, creating a honeycomb architecture with an average diameter of approximately 120 nm. The magnified image shown in Supplementary Figure S2 indicates that the particle size results are consistent with those in Figure 2C. The morphology of the honeycomb-like MnO_2_ nanospheres remained unchanged after 30 min of ultrasonic treatment, indicating that the nanospheres had high structural stability (Supplementary Figure S3). The UV-visible absorption spectrum showed that the main peaks of KMnO_2_ (319, 526 and 544 nm) disappeared when the reaction ended, with a new broad peak appearing at about 384 nm (Figure 2D), indicating the formation of a new species [26]. As shown in Figure 2E and F, TEM analysis revealed the morphology and size distribution of Liposome@Ica nanoparticles. The nanoparticles are nanoscale in size and have a spherical structure with a uniform size distribution, and the mean diameter of the Liposome@Ica nanoparticles was measured using a Malvern laser particle size analyzer and was 87.38 nm. In addition, zeta potential analysis showed that both MnO_2_ nanoparticles and liposomes exhibited negative surface charges (Supplementary Figure S4), further characterizing their surface electrostatic properties and dispersion behavior. Fourier transform infrared spectroscopy analysis was performed to verify amide bond formation between 4-arm PEG-NHS and the amino groups of SF in the PEG–fibroin hydrogel. After the formation of PEG-fibroin hydrogels, the characteristic C=O stretching vibration peak (1741 cm^−1^) of N-hydroxysuccinimide in 4-arm-PEG-NHS disappeared, whereas a new peak appeared at 1538 cm^−1^ (Figure 2G). The observed peak is associated with N–H bending vibrations characteristic of amide bonds. These results support our hypothesis that PEG-fibroin hydrogels are crosslinked via amide bonds [27]. Structural architecture is a fundamental determinant of hydrogel performance in tissue engineering applications. High water content and porous structures facilitate cell adhesion and proliferation, as well as tissue growth and differentiation. Following freeze-drying, the surface morphology of the hydrogels was examined by SEM. The hydrogel exhibited interconnected and uniformly distributed porous architecture with pore sizes ranging from 10 to 20 µm, facilitating cell growth and nutrient exchange (Figure 2H–J). As revealed by EDS analysis, MnO_2_ and Liposome@Ica nanoparticles were evenly distributed within the hydrogel network (Figure 2K). In addition, the FG/Mn@Ica hydrogels exhibited excellent injectability, enabling the *in situ* formation of homogeneous hydrogels. This superior injectability allows the material to conformally fill irregular microdefects and penetrate microwounds, thereby minimizing surgical trauma and facilitating minimally invasive implantation (Supplementary Figure S5).

**Figure 3:**
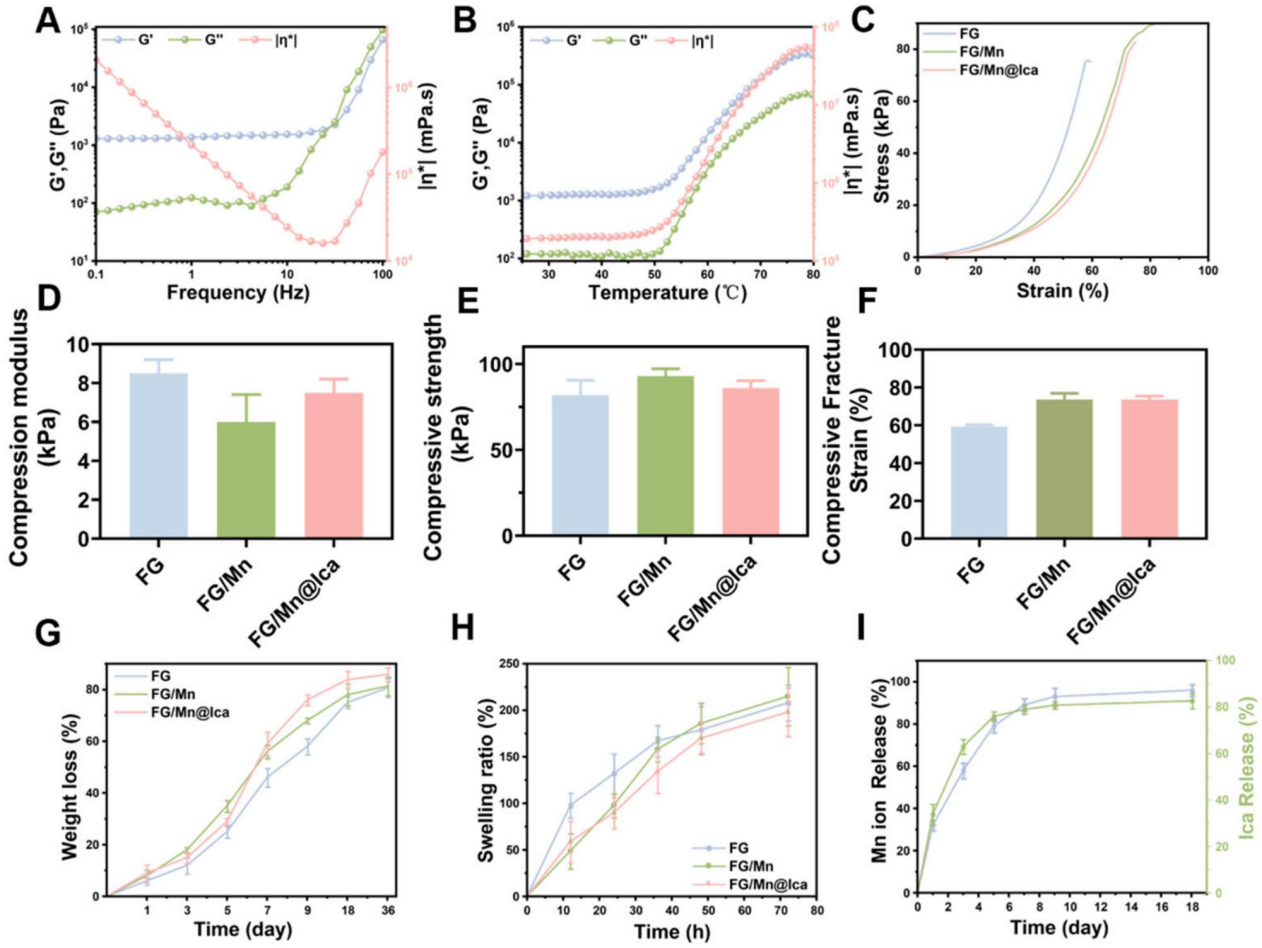
Evaluation of the mechanical properties and degradability of the FG/Mn@Ica hydrogel. (**A** and **B**) Rheological profiles of the FG/Mn@Ica hydrogels under frequency and temperature sweep modes. (**C**) Compressive stress-strain curves. (**D**–**F**) Quantitative analysis of the compressive modulus, strength, and fracture strain. (**G**) Degradation behavior. (**H**) Swelling ratio. (**I**) Cumulative release of Mn ions and Ica.

### Mechanical properties and degradability characterization of the FG/Mn@Ica hydrogel

To evaluate the mechanical properties of the gelled hydrogel and potential noncovalent interactions within the hydrogel network, the complex viscosity (η*), storage modulus (G′) and loss modulus (Gʺ) were determined, and compression tests were performed. The FG/Mn@Ica hydrogel had G′ > Gʺ at low frequencies and a crossover point between G′ and Gʺ at high frequencies, with G′ dominating across most frequency ranges, indicating elastic characteristics (Figure 3A). This crossover point signified a transition from the gel state to the sol state. The increase in viscous behavior was attributed to the disruption of hydrogen bond networks within the hydrogel, leading to greater energy dissipation. As shear rate increased, the viscosity decreased, indicating a typical shear-thinning pattern. This behavior originated from shear-induced molecular chain disentanglement and the weakening of intrachain and interchain interactions [14, 32]. The temperature-scanning test data are shown in Figure 3B. G′ dominated at all temperatures without crossing Gʺ, indicating that the hydrogel existed in a highly ordered, stable structural state. MnO_2_ incorporation might have increased both the G′ and G″ values across different temperatures [32]. These results demonstrate that the dynamic structure of the FG/Mn@Ica hydrogel has excellent stability and can sustain a porous network over extended periods. This provides an appropriate 3D microenvironment for intercellular interactions, promoting cell adhesion, migration and proliferation. Moreover, its viscoelastic properties mimic the mechanical characteristics of the bone extracellular matrix, effectively regulating cell fate and supporting the self-organization of organoids, thereby enhancing bone integration and remodeling [33].

**Figure 4:**
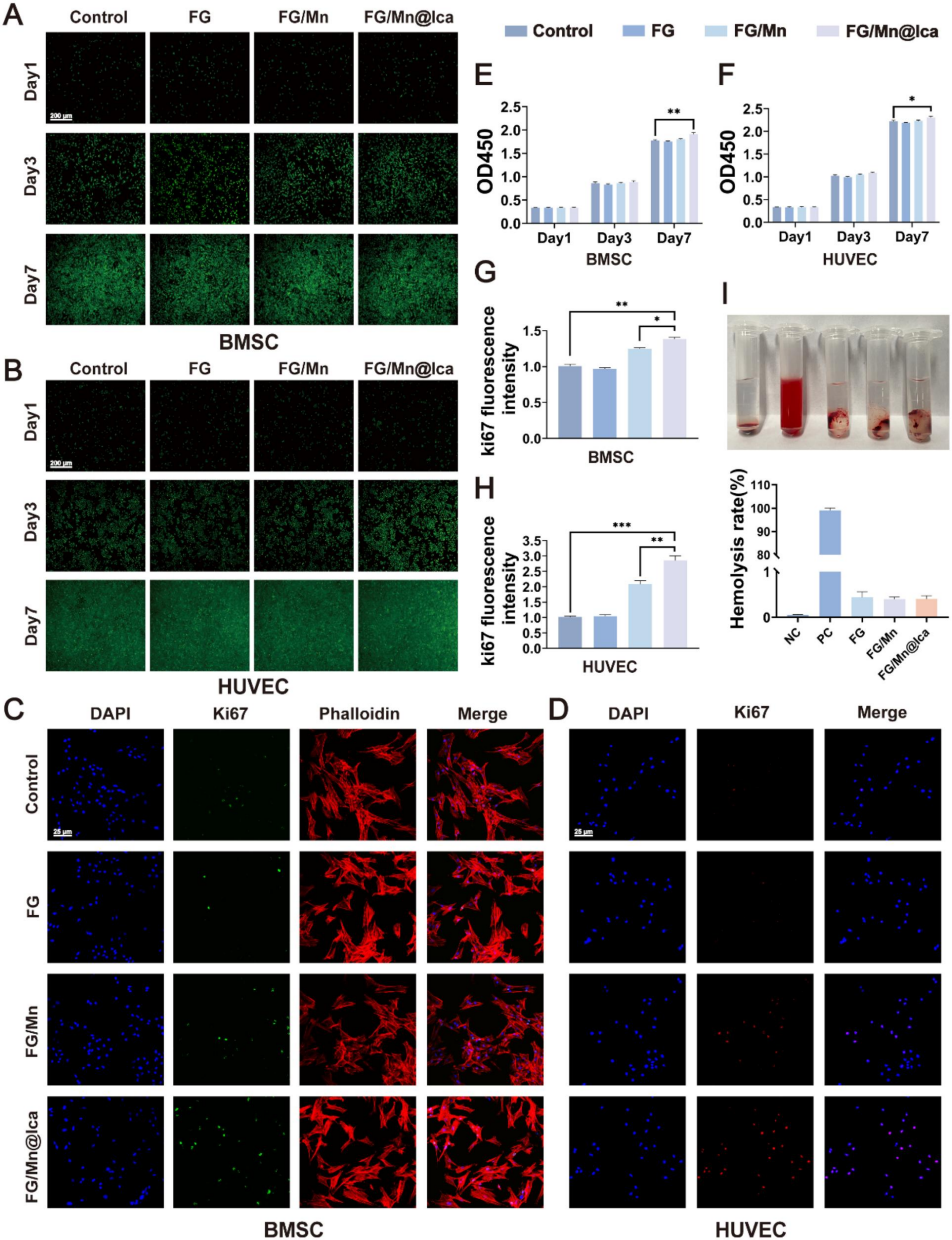
Cytocompatibility evaluation of the FG, FG/Mn, and FG/Mn@Ica hydrogels. (**A**) Live/dead staining of BMSCs after 1, 3 and 7 days of culture. (**B**) Live/dead staining of HUVECs at the corresponding time points. (**C** and **D**) Immunofluorescence staining of Ki-67 in BMSCs and HUVECs. (**E** and **F**) CCK-8 assays of BMSCs (**E**) and HUVECs (**F**) proliferation in response to different hydrogel treatments. (**G** and **H**) Quantitative analysis of Ki-67-positive cell ratios. (**I**) The hemolysis test results showed that all hydrogels exhibited hemolysis ratios < 5%. ***P < 0.001, **P < 0.01, *P < 0.05, *n* = 3.

Bone defect-filling materials typically require adequate mechanical properties to accommodate compression from surrounding tissues. The compression stress-strain test results (Figure 3C–F) showed that the FG hydrogel had a compressive strength of approximately 82 kPa, whereas the composite FG/Mn hydrogel had an enhanced compressive strength of 93 kPa. Compared to the FG hydrogel, the FG/Mn@Ica hydrogel had greater compressive strength (∼87 kPa) but a slightly lower compressive modulus, both of which were found to be suitable for bone repair applications. The compressive fracture strains of all components of the hydrogel ranged from 60% to 75%, further demonstrating the dynamic structural stability of the FG/Mn@Ica hydrogel. Following *in vivo* implantation, its mechanical strength increased adaptively and continuously, enabling support of the bone defect area and inducing osteogenic differentiation of BMSCs. Given its compressive strength in the tens-of-kPa range, this hydrogel is intended primarily for bone defect filling and local microenvironment modulation rather than for direct load-bearing support [34].

Biodegradability is a critical requirement for biomaterials intended for biomedical applications, as long-term persistence of hydrogels used for bone repair may cause adverse effects. The three different-component hydrogels exhibited similar degradation behavior, with mass loss rates ranging between 76% and 85% within 30 days (Figure 3G). The controlled degradation of the hydrogels was attributed to the crosslinked network structure formed by PEG and SF, which included amide bonds and hydrogen bonds, as illustrated in Figure 3E. The breakage of a few hydrogen bonds did not compromise the entire hydrogel network. When the number of broken hydrogen bonds and amide bonds reached a certain threshold, the entire crosslinked network gradually disassembled, leading to the progressive degradation of the hydrogel [34]. Excessive swelling of hydrogels can compress surrounding normal tissues or organs, causing localized damage [35]. Therefore, we evaluated the swelling rate of the FG/Mn@Ica hydrogel upon contact with water. The swelling rates of the three different hydrogels reached about 200 ± 25% after 3 days of immersion in water (Figure 3H). Consequently, these results indicate that the FG/Mn@Ica hydrogel will not exert excessive swelling forces on surrounding tissues when it is used in the moist environment of the body.

During degradation, the hydrogel gradually released bioactive components, including functional nanoparticles and therapeutic agents, which are essential for bone tissue regeneration. With the degradation of the FG/Mn@Ica hydrogel, MnO_2_ nanoparticles are gradually released into the tissue defect site, where they exert their biological effects (Figure 3I). Notably, approximately 90% of MnO_2_ was released by day 10, suggesting a pronounced early-stage antioxidant and microenvironment-regulating effects. To further directly verify the intrinsic antioxidative activity of MnO_2_ at the material level, its ROS-scavenging capability was evaluated by measuring the residual H_2_O_2_ after incubation with MnO_2_ nanoparticles for different periods. The results showed that MnO_2_ efficiently eliminated H_2_O_2_, and a concentration of 1 µg/mL removed nearly 70% of H_2_O_2_ within 2 h (Supplementary Figure S6), confirming its potent ROS-scavenging activity. Icariin was found to coexist within and outside the hydrogel as well as within the lipid matrix. It exhibited rapid release efficiency in the early stage and slower release efficiency in the later stage, with a release rate below 85% by day 10. The sustained release subsequently enhanced osteoblast function and bone formation. These results indicated that the FG/Mn@Ica hydrogel has adequate mechanical strength, excellent biodegradability and the capacity for sustained slow release of active factors, making it an ideal choice for bone repair [36].

### Formulation optimization and biocompatibility evaluation of FG/Mn@Ica hydrogels

To determine the appropriate concentrations of MnO_2_ and icariin (Ica) incorporated into the hydrogel, preliminary concentration-gradient optimization experiments were performed prior to the main biological evaluations. For MnO_2_, four different concentrations were first screened by assessing cell viability using the CCK-8 assay on days 1, 3 and 7 (Supplementary Figure S7, left). In addition, intracellular ROS levels in BMSCs were evaluated by DCFH-DA fluorescence staining, and the expression of osteogenic-related proteins, including OPN, RUNX2 and OCN, was analyzed by Western blotting after treatment with hydrogel extracts containing different MnO_2_ concentrations (Supplementary Figure S8). Based on the overall performance in promoting cell viability, reducing oxidative stress, and enhancing osteogenic differentiation, 0.25 mg/mL MnO_2_ was selected as the optimal concentration for subsequent experiments. After fixation of the MnO_2_ content at 0.25 mg/mL, different concentrations of Ica were further screened using a similar strategy. Specifically, cell viability was evaluated by CCK-8 assay on days 1, 3 and 7 (Supplementary Figure S7, right), while intracellular ROS levels and osteogenic protein expression were assessed by DCFH-DA staining and Western blotting, respectively (Supplementary Figure S9). The results showed that 0.5 mg/mL Ica achieved the best overall balance among cytocompatibility, antioxidative capacity and osteogenic activity without inducing detectable cytotoxicity. Therefore, 0.25 mg/mL MnO_2_ and 0.5 mg/mL Ica were selected as the final concentrations for incorporation into the FG/Mn@Ica hydrogel.

Biocompatibility is a primary prerequisite for the safe and effective application of biomaterials in tissue repair [37]. To assess the biocompatibility of the fabricated hydrogels, live/dead staining was performed on BMSCs and HUVECs cultured with the FG, FG/Mn and FG/Mn@Ica hydrogels for 1, 3 and 7 days (Figure 4A and B). In all groups, cell viability was high with negligible dead cells, indicating good cytocompatibility of the hydrogel systems. The number of viable cells increased with culture time, and a considerably denser distribution was observed in the FG/Mn@Ica group, implying that the incorporation of Mn ions and Ica provided a more favorable microenvironment for the adhesion and proliferation of cells. Immunofluorescence staining of Ki-67, a nuclear proliferation marker, further verified that the proliferative activity increased (Figure 4C and D). Compared to those cultured on FG or FG/Mn, those cultured on FG/Mn@Ica presented a substantially higher proportion of Ki-67-positive nuclei. Quantitative analysis confirmed a significant increase in Ki-67 expression in the FG/Mn@Ica group (Figure 4G and H), which was consistent with the CCK-8 assay results. The proliferation behavior was further evaluated by a CCK-8 assay on days 1, 3 and 7 (Figure 4E and F). Both BMSCs and HUVECs exhibited continuous growth, and the FG/Mn@Ica hydrogel induced the highest optical density (OD) values at all time points. These results suggest that the combined action of Mn ions and Ica significantly promoted cellular metabolic activity and proliferation. The hemocompatibility of the hydrogels was confirmed by hemolysis testing (Figure 4I). All samples presented hemolysis ratios far below the 5% threshold defined for biomedical materials, demonstrating excellent blood compatibility [38]. Collectively, these findings confirmed that the FG/Mn@Ica hydrogel has greater cytocompatibility and hemocompatibility compared to those of FG and FG/Mn, providing a safe and supportive microenvironment for osteogenic (BMSCs) and angiogenic (HUVECs) cells and establishing a solid biological foundation for subsequent antioxidative and osteogenic investigations.

**Figure 5:**
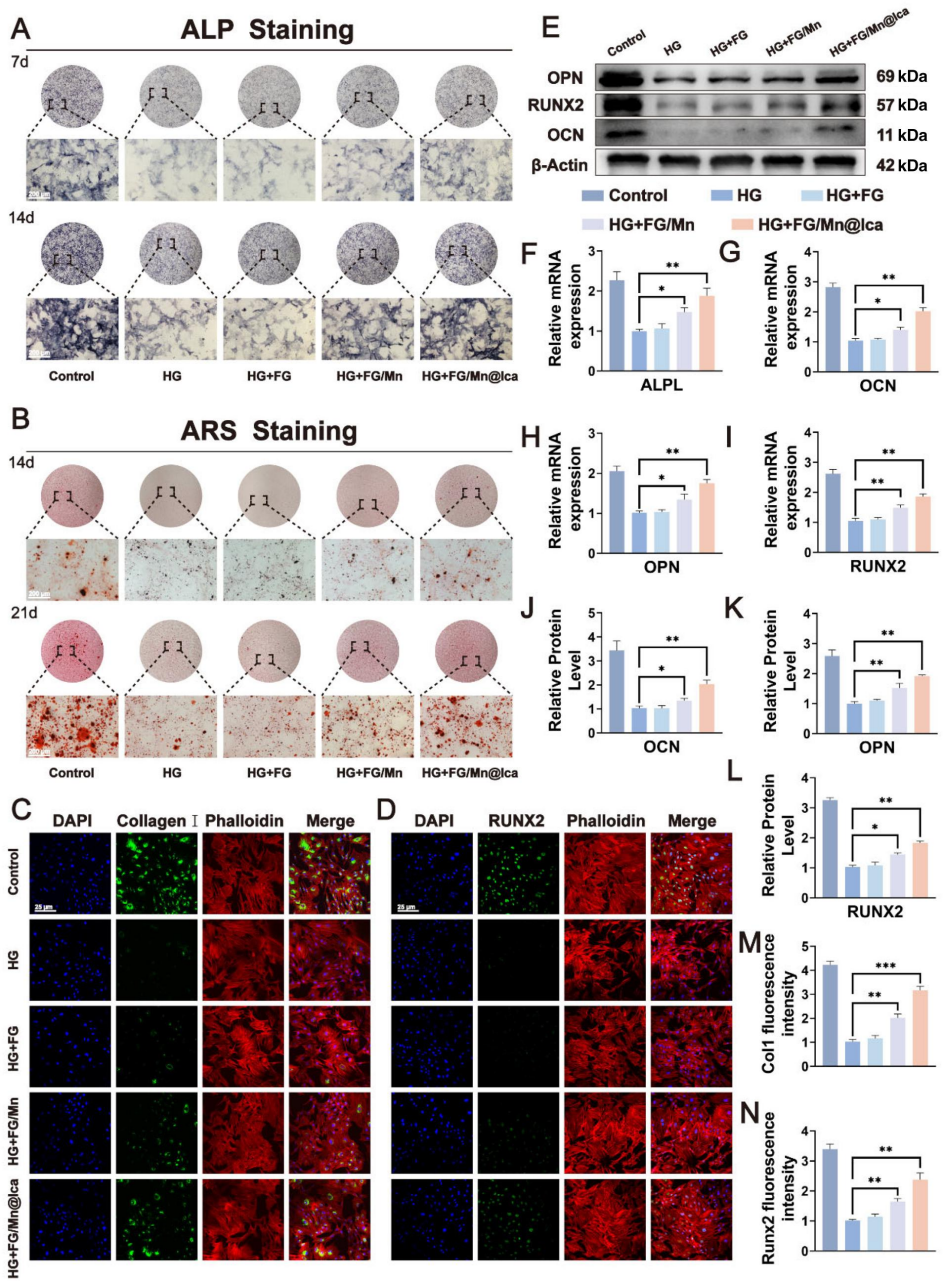
Osteogenic differentiation of BMSCs cultured with the FG, FG/Mn, and FG/Mn@Ica hydrogels. (**A**) ALP staining performed at days 7 and 14. (**B**) ARS staining conducted at days 14 and 21. (**C** and **D**) Immunofluorescence staining of Collagen I and RUNX2. (**E**) Western blotting analysis of OCN, OPN and RUNX2 expression. (**F**–**I**) Quantitative RT-qPCR analysis of ALPL, OCN, OPN and RUNX2 mRNA expression. (**J**–**L**) Western blotting quantification of OCN, OPN and RUNX2 protein levels. (**M** and **N**) Quantitative fluorescence analysis of Collagen I and RUNX2 immunostaining. ***P < 0.001, **P < 0.01, *P < 0.05, *n* = 3.

### Enhanced osteogenic differentiation and matrix mineralization of BMSCs induced by FG/Mn@Ica hydrogels

After confirming the excellent cytocompatibility of the hydrogels, their osteogenic potential was further investigated using BMSCs cultured under HG conditions to mimic the diabetic microenvironment *in vitro* [39]. ALP was used as an early osteogenic marker, whereas ARS staining was applied to evaluate matrix mineralization at later stages [40, 41]. ALP staining at days 7 and 14 revealed a time-dependent increase in ALP activity across all groups, with the FG/Mn@Ica group exhibiting the most intense staining, indicating enhanced early osteogenic differentiation (Figure 5A). Similarly, ARS staining on days 14 and 21 (Figure 5B) revealed markedly denser calcium nodule deposition in the FG/Mn@Ica group than in the FG and FG/Mn groups, suggesting enhanced matrix mineralization. Immunofluorescence staining of Collagen I and RUNX2 (Figure 5C, D, M, N) further supported these findings. BMSCs treated with FG/Mn@Ica presented an increase in the expression of collagen I, reflecting increased synthesis of the extracellular matrix, and RUNX2, a key osteogenic transcription factor, indicated an increase in osteogenic activation [42, 43]. To verify the expression of osteogenesis-related markers at the molecular level, Western blotting and RT-qPCR analyses were conducted. The protein levels of OPN, RUNX2 and OCN were substantially increased in the FG/Mn@Ica group (Figure 5E). Densitometric quantification (Figure 5J–L) confirmed that the expression of these genes was significantly greater than that of FG and FG/Mn. Consistently, the mRNA levels of ALPL, OPN, RUNX2, and OCN increased significantly (Figure 5F-I), indicating transcriptional activation of osteogenic genes. Collectively, these results indicated that the synergistic incorporation of Mn ions and Ica effectively enhances both the early and late stages of the osteogenic differentiation of BMSCs. Mn, as a trace element related to bone metabolism, may mainly provide microenvironmental and metabolic support for osteogenesis, whereas Ica may more directly promote osteogenic differentiation and the expression of osteogenic markers [24, 44]. This complementary effect may partly explain the superior osteogenic activity of the composite hydrogel.

**Figure 6:**
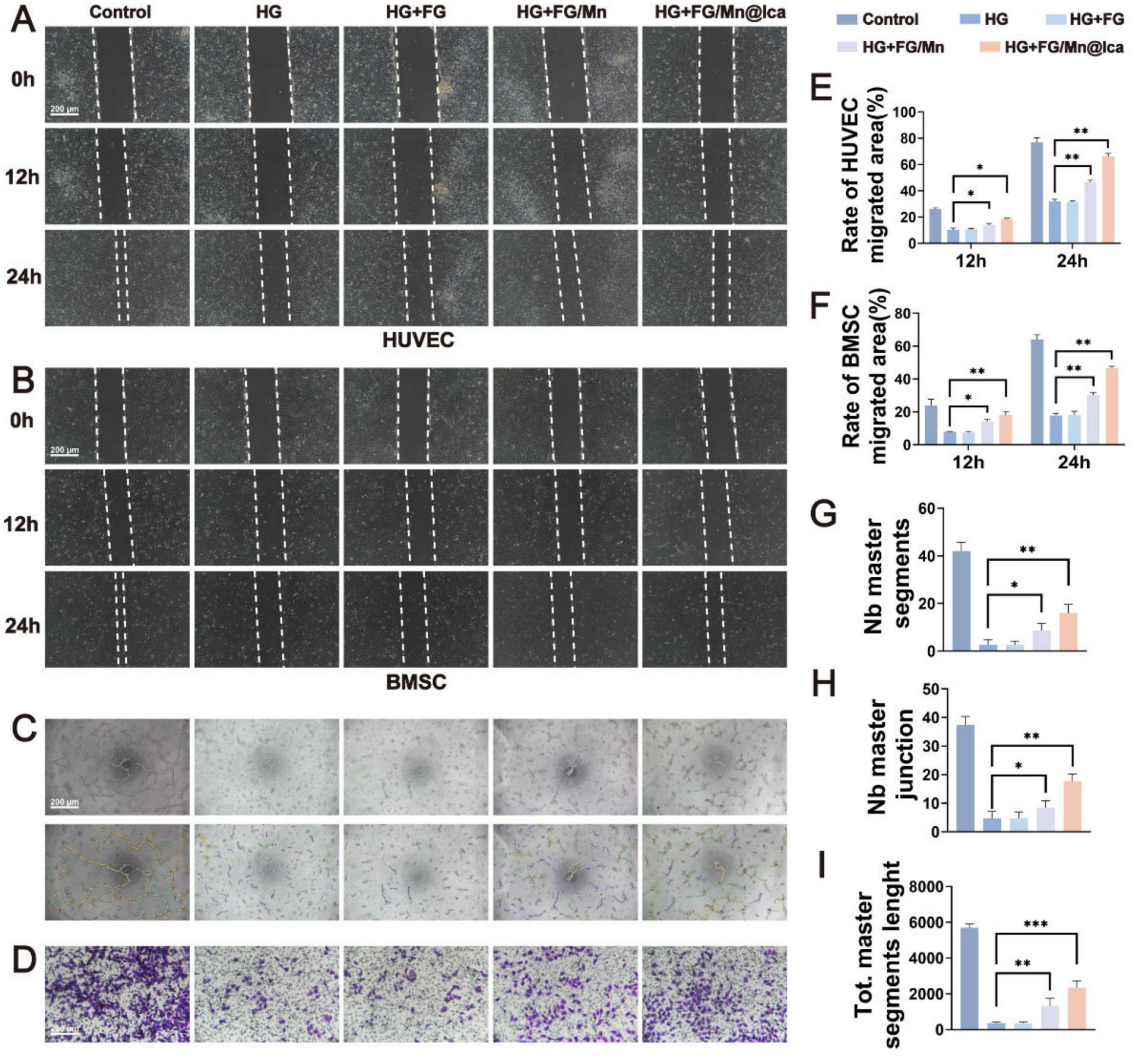
Cell migration and angiogenesis evaluation under high-glucose conditions. (**A** and **B**) Scratch wound healing assays of HUVECs and BMSCs at 0, 12 and 24 h. (**C**) Tube formation of HUVECs after 12 h of culture. (**D**) Transwell migration of HUVECs. (**E** and **F**) Quantitative analysis of the migration of HUVECs and BMSCs. (**G**–**I**) Quantification of angiogenic parameters. ***P < 0.001, **P < 0.01, *P < 0.05, *n* = 3.

### FG/Mn@Ica hydrogel enhances cell migration and angiogenic capacity under HG conditions

In diabetic bone defects, angiogenesis is particularly important because endothelial dysfunction and insufficient neovascularization are major barriers to effective bone healing [3]. Impaired endothelial migration and tube-forming capacity hinder vascular network formation, thereby limiting the establishment of a favorable regenerative microenvironment [45]. Therefore, promoting angiogenesis is essential not only for vascular reconstruction but also for supporting subsequent osteogenesis and bone repair [46–48]. We further evaluated the regenerative potential of the FG/Mn@Ica hydrogels under oxidative stress conditions by conducting wound healing and angiogenesis assays on BMSCs and HUVECs cultured in HG media. Both HUVECs and BMSCs in the FG/Mn@Ica group displayed significantly faster wound closure at 12 and 24 h than those in the FG and FG/Mn groups (Figure 6A and B), indicating an increase in migratory activity even in the hyperglycemic environment. Quantitative analysis (Figure 6E and F) confirmed that the migration rates of HUVECs and BMSCs in the FG/Mn@Ica group were considerably greater than those in the other groups. The results of the Transwell assay (Figure 6D) further validated these findings, as more cells migrated through the membrane after FG/Mn@Ica treatment, supporting the strong chemotactic and motility-promoting effects of FG/Mn@Ica. To evaluate angiogenic capability, HUVECs were used to perform tube formation assays (Figure 6C). The FG/Mn@Ica group exhibited a well-organized and dense capillary-like network, whereas the FG and FG/Mn groups formed sparse or incomplete structures. Quantitative analysis of total tube length, branch points, and mesh area (Figure 6G–I) revealed a significant increase in the FG/Mn@Ica group, confirming the synergistic proangiogenic effect of Mn ions and Ica. These results indicated that the FG/Mn@Ica hydrogel not only rescues cell motility impaired by hyperglycemia but also robustly promotes endothelial angiogenesis, which may facilitate neovascularization and tissue regeneration in diabetic bone defects.

**Figure 7:**
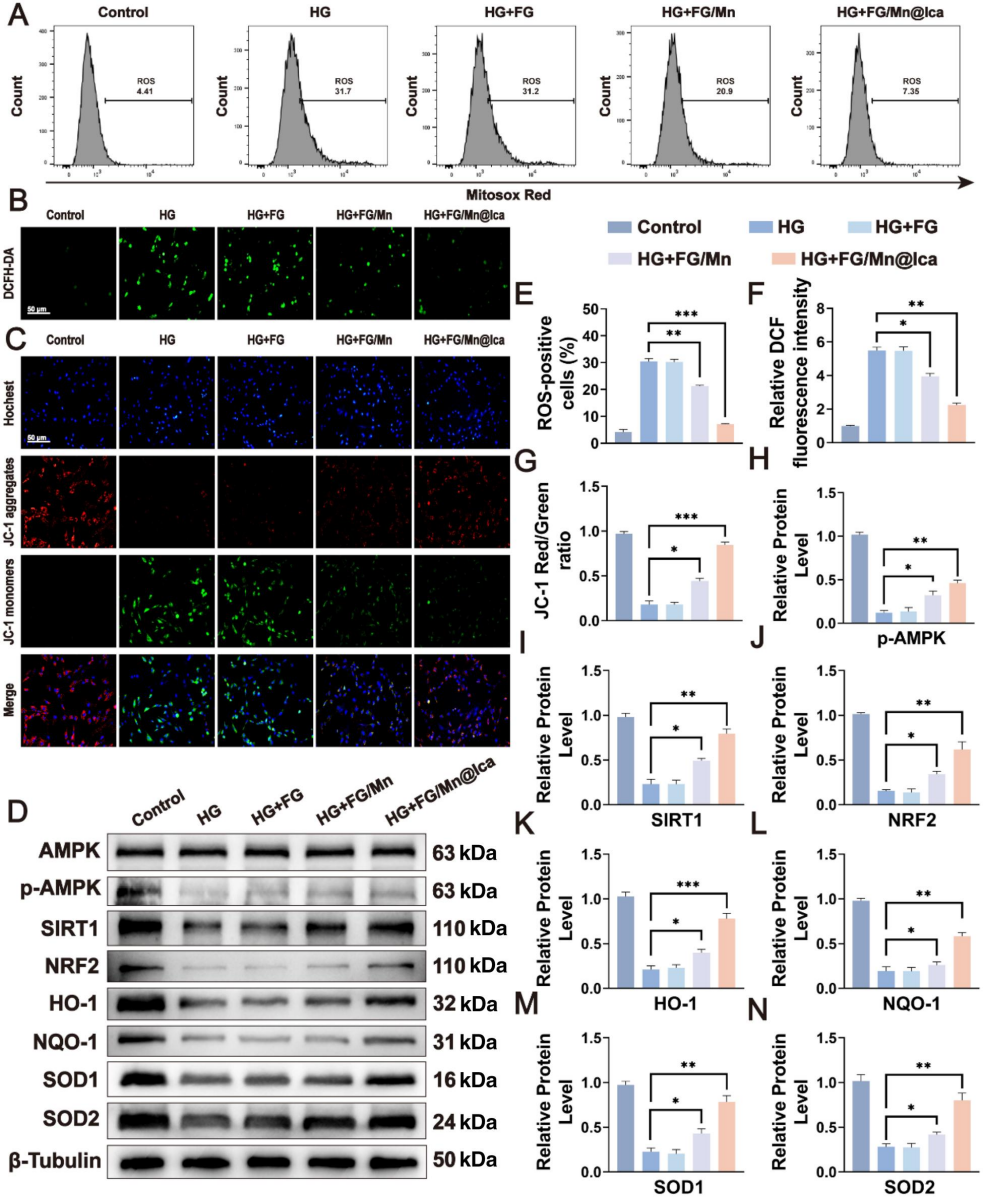
The FG/Mn@Ica hydrogel activates AMPK–SIRT1–NRF2 signaling and relieves oxidative stress in HG-treated BMSCs. (**A**) Flow cytometry analysis of intracellular ROS levels was evaluated using the MitoSOX Red probe. (**B**) Representative fluorescence images of DCFH-DA stained BMSCs treated with various hydrogels. (**C**) JC-1 staining shows the mitochondrial membrane potential (ΔΨm). (**D**) Western blotting analysis was employed to determine the expression of AMPK, p-AMPK, SIRT1, NRF2, HO-1, NQO-1, SOD1, SOD2 and β-tubulin. (**E**–**G**) Quantitative analyses of ROS-positive cell ratios, DCF fluorescence intensity, and the JC-1 red/green ratio. (**H**–**N**) Densitometric quantification of the AMPK-SIRT1-NRF2 pathway and downstream antioxidant proteins. ***P < 0.001, **P < 0.01, *P < 0.05, *n* = 3.

### FG/Mn@Ica hydrogel attenuates oxidative stress and inflammatory signaling in BMSCs under HG conditions

As diabetic bone defects are frequently accompanied by excessive ROS generation and redox imbalance, leading to oxidative injury and impaired cellular function, we next sought to clarify the intracellular mechanisms by which the FG/Mn@Ica hydrogel mitigates HG-induced damage [49, 50]. Accordingly, we assessed oxidative stress levels, mitochondrial function, and antioxidant signaling in BMSCs, since sustained accumulation of ROS can damage cellular membranes and DNA and ultimately induce cell death [51]. Flow cytometry analysis was performed using the MitoSOX Red probe (Figure 7A), and the results revealed that BMSCs exposed to high glucose presented pronounced ROS accumulation. In contrast, cells cultured with the FG/Mn or FG/Mn@Ica hydrogel presented progressively reduced ROS signals, with the FG/Mn@Ica group exhibiting the lowest fluorescence intensity. The fluorescence microscopy images (Figure 7B) were consistent with these observations, showing significantly weaker green fluorescence after treatment with the dual-functional hydrogel. As shown in Figure 7E and F, FG/Mn@Ica group treatment reduced intracellular ROS levels by more than 60% relative to the HG control, indicating strong ROS-scavenging capacity. Given the association between oxidative stress and mitochondrial impairment, the mitochondrial membrane potential (ΔΨm) was next examined using JC-1 staining (Figure 7C). HG treatment induced a loss of ΔΨm, reflected by a prominent shift from red J-aggregates to green monomers. The FG/Mn hydrogel partially restored the red signal, whereas the FG/Mn@Ica treatment considerably increased the red/green ratio, indicating that mitochondrial integrity was preserved and depolarization decreased. Quantitative analysis (Figure 7G) revealed significant restoration of the ΔΨm ratio relative to that in the HG group, suggesting that the hydrogel effectively preserved the mitochondrial potential and maintained cellular energy homeostasis. To further elucidate the molecular mechanism underlying these protective effects, Western blotting assays were performed with densitometric analysis (Figure 7D, H–N). The results revealed that exposure to high glucose markedly suppressed the phosphorylation of AMPK and reduced the expression of SIRT1 and NRF2, together with a decrease in their downstream antioxidant enzymes, including HO-1, NQO-1, SOD1 and SOD2. Functionally, AMPK serves as a central energy-sensing kinase that maintains metabolic homeostasis; SIRT1 regulates mitochondrial and stress-responsive transcriptional programs; NRF2 serves as a master antioxidant transcription factor; and HO-1/NQO-1 and SOD1/SOD2 constitute key enzymatic effectors responsible for ROS detoxification and redox balance [52–56]. Thus, the collective downregulation of these protective molecules under hyperglycemic stress reflects a shift toward a pro-oxidative and injury-susceptible intracellular state. In contrast, FG/Mn and particularly FG/Mn@Ica reversed these alterations, restoring the phosphorylation of AMPK and enhancing the expression of SIRT1, NRF2, and their downstream antioxidant enzymes. Among all the groups, the FG/Mn@Ica group elicited the most robust activation of the AMPK-SIRT1-NRF2 axis and upregulation of its effectors, indicating the reinforcement of intracellular redox defense and metabolic resilience. MnO_2_ may primarily exert its antioxidative effect through direct ROS scavenging [57]. In contrast, Ica may further enhance cellular antioxidant defense and functional recovery, potentially by promoting the transcription of antioxidant-related genes [58]. These complementary, and to some extent overlapping, roles in antioxidative regulation may explain the synergistic therapeutic effect of the composite hydrogel. This coordinated signaling cascade alleviates ROS overload, preserves mitochondrial function, and attenuates secondary inflammatory injury. Overall, FG/Mn@Ica reprograms the responses of BMSCs toward a protective, low-oxidative, and energy-balanced state through dual reinforcement of AMPK–SIRT1 signaling and NRF2-mediated antioxidant gene expression, providing a molecular basis that links its antioxidative regulation to the enhanced osteogenic outcomes observed *in vivo*.

**Figure 8:**
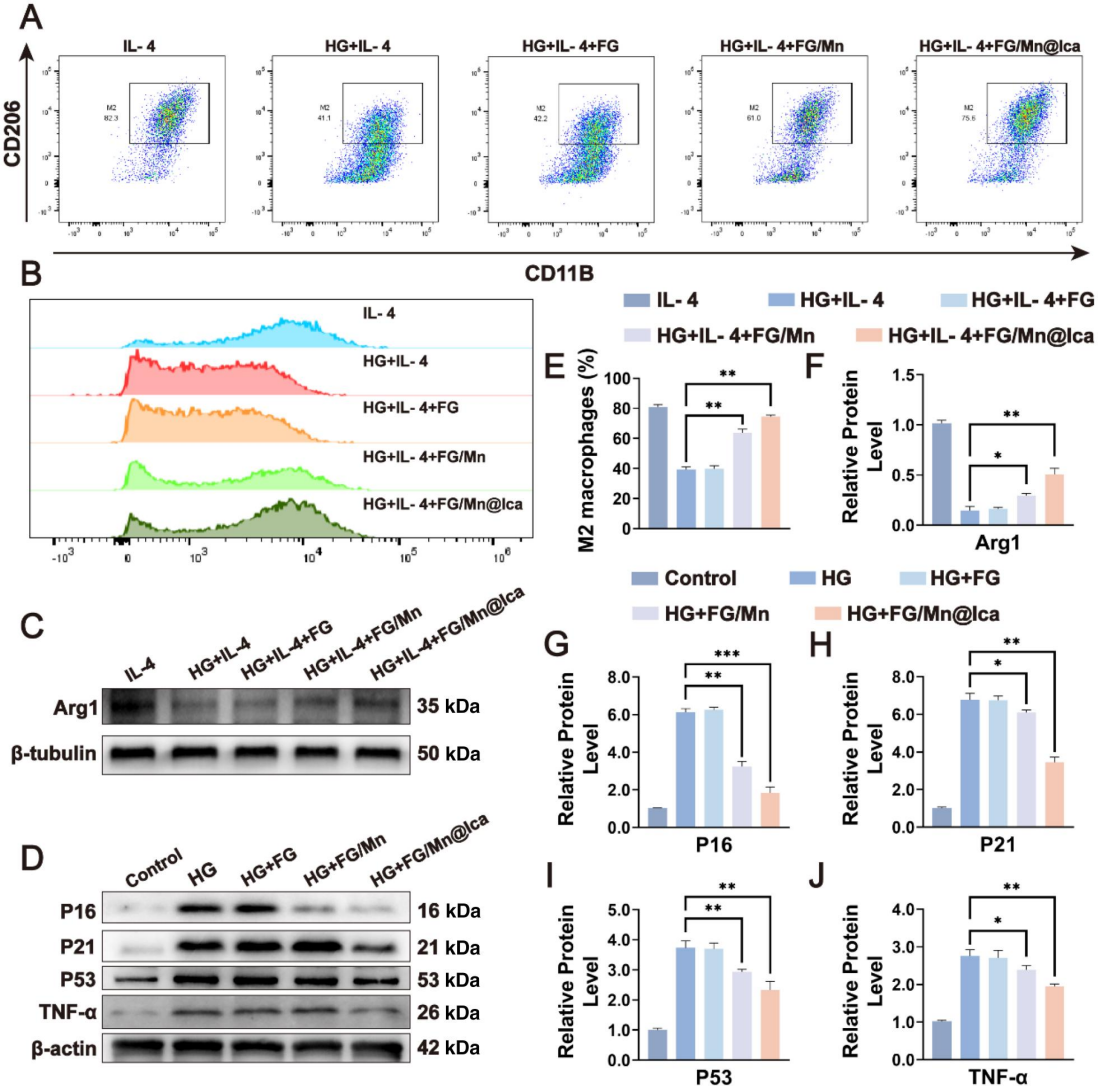
FG/Mn@Ica Modulates macrophage polarization and attenuates senescence-associated inflammation. (**A**) Flow cytometry analysis of macrophage polarization. (**B**) Overlay histogram of the flow cytometry results. (**C**) Western blotting analysis of Arg1 in BMDMs. (**D**) Western blotting analysis of P16, P21, P53 and TNF-α in BMSCs. (**E**) Quantification of the proportion of M2 macrophages. (**F**–**J**) Densitometric analysis of the expression of Arg1, P16, P21, P53 and TNF-α. ***P < 0.001, **P < 0.01, *P < 0.05, *n* = 3.

### FG/Mn@Ica regulates macrophage polarization and suppresses senescence-associated inflammation under HG conditions

Immune regulation plays a pivotal role in diabetic bone regeneration [59]. Accordingly, we further evaluated the effects of FG/Mn@Ica on macrophage polarization and senescence-associated inflammatory responses. To establish a macrophage polarization model, BMDMs were treated with IL-4, a classical cytokine widely used to induce M2 polarization. This model was used to determine whether the hydrogel could further promote a pro-regenerative and anti-inflammatory macrophage phenotype [60]. Flow cytometry analysis of BMDMs (Figure 8A and E) revealed that, compared to the FG and FG/Mn groups, the FG/Mn@Ica treatment markedly increased the proportion of M2-like macrophages, a pro-regenerative and anti-inflammatory phenotype [61]. Consistently, overlay histograms revealed a pronounced increase in CD206 fluorescence intensity in the FG/Mn@Ica group (Figure 8B), indicative of enhanced M2-associated surface marker expression. To confirm macrophage polarization at the protein level, the expression of Arginase-1 (Arg1), a canonical M2 macrophage marker, was examined by Western blotting analysis [62]. The expression of Arg1 was substantially upregulated in the FG/Mn@Ica group (Figure 8C and F), which provided additional evidence of a shift toward an anti-inflammatory M2 phenotype. Given that hyperglycemic stress is closely associated with cellular senescence and chronic inflammation, we next assessed senescence-related and inflammation-related markers in BMSCs. The results of Western blotting assays (Figure 8D) demonstrated that the expression of senescence-associated proteins P16, P21 and P53, together with the pro-inflammatory cytokine TNF-α, was markedly increased under HG conditions, whereas FG/Mn@Ica treatment effectively suppressed these proteins. Corresponding quantitative analysis (Figure 8G–J) confirmed the significant downregulation of senescence-associated and inflammatory markers, indicating that FG/Mn@Ica alleviates stress-induced cellular aging and inflammatory signaling. Overall, these findings revealed that FG/Mn@Ica not only promotes an anti-inflammatory, M2-dominant macrophage phenotype but also mitigates senescence-associated inflammation in BMSCs, thereby contributing to the establishment of a pro-regenerative immune microenvironment under diabetic conditions.

**Figure 9:**
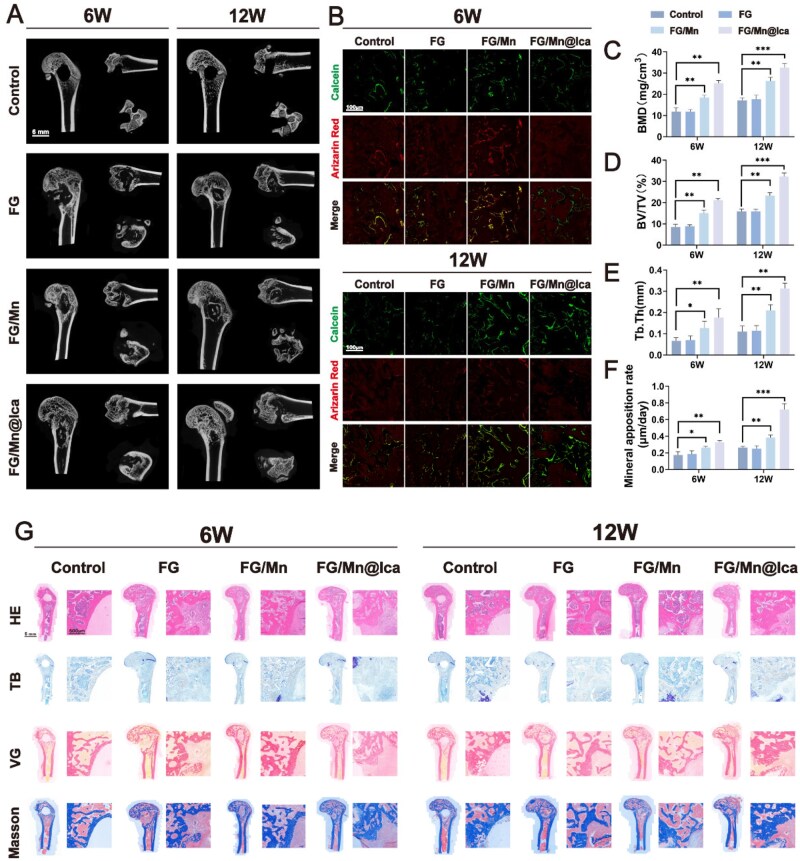
FG/Mn@Ica enhances bone regeneration in a diabetic femoral defect model. (**A**) 3D micro-CT reconstructions of femoral defects were obtained 6 and 12 weeks after implantation. (**B**) Sequential fluorochrome labeling was performed, illustrating calcein-labeled and alizarin-labeled mineral deposition at 6 and 12 weeks. (**C**–**E**) Quantitative micro-CT-derived parameters, including BMD, BV/TV and Tb.Th, are presented. (**F**) Mineral apposition rate (MAR) measurement. (**G**) Histological evaluation of femoral defect sections at 12 weeks was performed after H&E, TB, VG and Masson staining. ***P < 0.001, **P < 0.01, *P < 0.05, *n* = 3.

### FG/Mn@Ica significantly enhances bone regeneration in a diabetic femoral defect model

To evaluate the *in vivo* bone regenerative capacity of the hydrogels, a previously established femoral condyle defect model was used, as described in previous studies [63, 64]. The selected time points (6 and 12 weeks) were chosen to represent distinct stages of bone healing in diabetic bone defects [23]. Briefly, 6 weeks was used as an intermediate endpoint to capture the early-to-mid stage of mineralized tissue formation and initial defect repair, whereas 12 weeks was selected as a later endpoint to evaluate more mature bone remodeling and structural consolidation, which is particularly important given that bone regeneration is often delayed under diabetic conditions. At 12 weeks post-implantation, blood biochemical and hematological analyses revealed no significant abnormalities, indicating good systemic biocompatibility of the implanted hydrogels (Supplementary Figure S10). 3D micro-CT reconstruction (Figure 9A) revealed limited new bone formation in the FG group at both 6 and 12 weeks, whereas FG/Mn treatment moderately increased bone ingrowth in the defect region. In contrast, the content of newly formed bone increased in the FG/Mn@Ica group at both time points, indicating an overall improvement in osteogenic activity, although complete defect bridging was not observed at 12 weeks. To further assess dynamic bone formation, sequential fluorochrome labeling was performed. Calcein and alizarin red labeling revealed sparse and discontinuous mineral deposition in the FG group, whereas FG/Mn treatment resulted in increased but still heterogeneous labeling (Figure 9B). The FG/Mn@Ica group displayed broader and more continuous double labeling, suggesting sustained mineral deposition over time. Quantitative analysis of the MAR (Figure 9F) revealed a significantly greater MAR in the FG/Mn@Ica group than in the FG and FG/Mn groups, indicating accelerated bone matrix deposition and active remodeling dynamics. Quantitative micro-CT evaluation (Figure 9C–E) confirmed these observations, indicating a marked increase in BMD, BV/TV and Tb.Th in the FG/Mn@Ica group at both 6 and 12 weeks. These changes reflect enhancements in bone quality and trabecular microarchitecture rather than complete defect bridging. Consistent with the radiographic results, histological analyses (Figure 9G) revealed more pronounced new bone formation and collagen deposition in the FG/Mn@Ica group, as indicated by H&E, toluidine blue, van Gieson, and Masson’s trichrome staining. In contrast, the FG group predominantly exhibited fibrous tissue within the defect region. Collectively, these results suggest improved bone formation and maturation *in vivo*, consistent with the increase in MAR and fluorochrome labeling outcomes.

## Conclusion

In this study, we engineered an injectable and biodegradable FG/Mn@Ica hydrogel that integrates structural support with coordinated biochemical regulation to promote bone regeneration under hyperglycemic conditions. The hydrogel featured a porous, elastic-dominant, and mechanically adaptive network with controlled degradability and sustained release of Mn ions and Ica, thereby reconstructing a stable and functionally instructive microenvironment. *In vitro*, FG/Mn@Ica not only enhanced the proliferation of BMSCs and osteogenic differentiation but also mitigated glucose-induced oxidative stress, restored mitochondrial homeostasis, activated the AMPK–SIRT1–NRF2 antioxidant axis, promoted the polarization of pro-regenerative M2 macrophages, and attenuated senescence-associated and inflammation-associated responses, collectively reprogramming the dysregulated osteoimmune-redox interface. *In vivo*, the hydrogel significantly promoted new bone regeneration in a rat femoral defect model. These findings collectively demonstrate that FG/Mn@Ica simultaneously integrates mechanical compatibility, restoration of redox homeostasis, osteoimmune modulation, and osteogenic reinforcement, highlighting a rational biomaterial strategy that targets the pathological microenvironment rather than osteogenesis alone and offering meaningful translational potential for bone defect repair in metabolically compromised conditions such as diabetes.

## Supplementary Material

rbag143_Supplementary_Data
