# Supplementary material for: Injectable Mn–Icariin-functionalized silk fibroin/PEG hydrogels restore redox homeostasis and reprogram osteogenic–angiogenic coupling for diabetic bone regeneration
Source: Regen Biomater. 2026 Jul 3;13:rbag143. doi: 10.1093/rb/rbag143 (PMC13437042; doi:10.1093/rb/rbag143)
Supplement: Supplementary file 1 [file rbag143_supplementary_data.zip › SI 终版.docx]

*Supplementary Information For*

**Injectable Mn–Icariin-Functionalized Silk Fibroin/PEG Hydrogels Restore Redox Homeostasis and Reprogram Osteogenic–Angiogenic Coupling for Diabetic Bone Regeneration**

**Authors：**

*Yu-Kai Huang^,a,^****^#^****, Shi-Hao Zhang^c,^****^#^****, Xiao-Gang Zhou^b,^****^#^*** *, Lang Qin ^c,^****^#^****, Zeng Lin ^b^, Bing-Huang Yao ^a^, Zhi-Yang Gao ^a^ , Huan-Xin He ^a^, Jin-Wang Huang ^d,e^, Jie Peng ^f^, Hui-Zhen Du ^b^, Ding-Xin Wang ^a^, Jun-Yuan Zeng ^a^, Di-Han Su ^b^ , Zeng-Gan Chen ^b,d,e,*^, Yu-Lin Li ^c,*^, Li-Bo Jiang ^b,d,e, *^, Ming-Dong Zhao^a,*^*

**Affiliations:**

*^a^ Department of Orthopaedics, Jinshan Hospital, Fudan University, Shanghai, 201508, P. R. China*

*^b^* *Department of Orthopaedic Surgery, Zhongshan Hospital, Fudan University, Shanghai, 200032, P. R. China*

*^c^ Key Laboratory for Ultrafine Materials of Ministry of Education, Frontiers Science Center for Materiobiology and Dynamic Chemistry, Research Center for Biomedical Materials of Ministry of Education, School of Materials Science and Engineering, East China University of Science and Technology, Shanghai, 200237, P. R. China*

*^d^ Department of Orthopedic Surgery, Zhongshan Hospital (Xiamen), Fudan University, Fujian, 361015, P. R. China*

*^e^ Xiamen Osteoporosis Specialized Prevention and Treatment Center, Xiamen, 361000, P. R. China*

*^f^ Clinical Medical College of Xiamen Medical College, Fujian, 361023, P. R. China*

***^#^*** These authors contributed equally: *Yu-Kai Huang, Shi-Hao Zhang, Xiao-Gang Zhou, Lang Qin*

*^*^Correspondence address. E-mail:* *zhao_mingdong@fudan.edu.cn (M-D.Z.); jiang.libo@zs-hospital.sh.cn (L-B.J.);* *yulinli@ecust.edu.cn (Y-L. L.); chen.zenggan@zs-hospital.sh.cn (Z-G.C.)*

**Figure S1.** Schematic diagram of MnO_2_ nanoparticle preparation.

**Figure S2.** TEM image of the MnO_2_ nanoparticles.

**Figure S3.** TEM image of MnO_2_ nanoparticles following ultrasonic treatment.

**Figure S4.** (A) Zeta potential of MnO_2_ nanoparticles and liposomes; (B) Phase diagram. (n = 3)

**Figure S5.** The hydrogel exhibited good plasticity and injectability, allowing it to be shaped and maintained as a stable bulk structure after gelation.

**Figure S6.** ROS-scavenging capability of MnO_2_ nanoparticles. (n = 3)

**Figure S7.** (Left) Cells were cultured with hydrogel extracts containing different concentrations of MnO₂, and cell viability was assessed at Days 1, 3, and 7 using the CCK-8 assay. (Right) Cells were cultured with hydrogel extracts containing different concentrations of icariin (with optimized MnO₂ content fixed), and cell viability was measured at Days 1, 3, and 7 using the CCK-8 assay. (***p < 0.001, **P < 0.01, *P < 0.05, n = 3)

**Figure S8.** (A) Western blot analysis of osteogenic-related proteins, including OPN, RUNX2, and OCN, after treatment with hydrogels containing different MnO₂ concentrations. (B) Intracellular reactive oxygen species (ROS) levels in BMSCs cultured with hydrogel extracts containing different MnO₂ concentrations were evaluated using DCFH-DA fluorescence staining. (n = 3)

**Figure S9.** (A) Western blot analysis of osteogenic markers, including OPN, RUNX2, and OCN, showing that an appropriate icariin concentration further promotes osteogenic differentiation under the optimized MnO₂ condition. (B) Intracellular ROS levels were detected using the ROS-sensitive fluorescent probe DCFH-DA in BMSCs treated with hydrogel extracts containing different icariin concentrations (under fixed optimized MnO₂ conditions). (n = 3)

**Figure S10.** Blood biochemical indicators of rats in 12 weeks after material implantation. (n = 3)
